# Implication of fibroblast growth factors in epileptogenesis-associated circuit rearrangements

**DOI:** 10.3389/fncel.2013.00152

**Published:** 2013-09-13

**Authors:** Beatrice Paradiso, Silvia Zucchini, Michele Simonato

**Affiliations:** ^1^Department of Medical Sciences, Section of Pharmacology, University of FerraraFerrara, Italy; ^2^Department of Morphology, Surgery and Experimental Medicine, Section of PathologyFerrara, Italy; ^3^National Institute of Neuroscience, University of FerraraFerrara, Italy; ^4^Laboratory of Technologies for Advanced Therapy (LTTA), Technopole of FerraraFerrara, Italy

**Keywords:** fibroblast growth factors, epilepsy, cell death, neurogenesis, synaptogenesis

## Abstract

The transformation of a normal brain in epileptic (epileptogenesis) is associated with extensive morpho-functional alterations, including cell death, axonal and dendritic plasticity, neurogenesis, and others. Neurotrophic factors (NTFs) appear to be very strongly implicated in these phenomena. In this review, we focus on the involvement of fibroblast growth factor (FGF) family members. Available data demonstrate that the FGFs are highly involved in the generation of the morpho-functional alterations in brain circuitries associated with epileptogenesis. For example, data on FGF2, the most studied member, suggest that it may be implicated both in seizure susceptibility and in seizure-induced plasticity, exerting different, and apparently contrasting effects: favoring acute seizures but reducing seizure-induced cell death. Even if many FGF members are still unexplored and very limited information is available on the FGF receptors, a complex and fascinating picture is emerging: multiple FGFs producing synergic or antagonistic effects one with another (and/or with other NTFs) on biological parameters that, in turn, facilitate or oppose transformation of the normal tissue in epileptic. In principle, identifying key elements in these phenomena may lead to effective therapies, but reaching this goal will require confronting a huge complexity. One first step could be to generate a “neurotrophicome” listing the FGFs (and all other NTFs) that are active during epileptogenesis. This should include identification of the extent to which each NTF is active (concentrations at the site of action); how it is active (local representation of receptor subtypes); when in the natural history of disease this occurs; how the NTF at hand will possibly interact with other NTFs. This is extraordinarily challenging, but holds the promise of a better understanding of epileptogenesis and, at large, of brain function.

## Introduction

Acquired epileptic syndromes are characterized by the spontaneous appearance of seizures in a previously healthy brain. Many acquired epilepsies have an identifiable cause, such as a head trauma, an episode of status epilepticus (SE), a stroke, or a brain infection (Pitkänen and Sutula, 2002). It is thought that these damaging insults set in motion a cascade of neurobiological alterations that, in time, will lead to the occurrence of spontaneous seizures and to the diagnosis of epilepsy. This phenomenon is termed “epileptogenesis”.

Conventional “antiepileptic” agents exert only symptomatic effects on seizures but do not interfere with epileptogenic processes (Temkin, 2001). Moreover, a third of the people with epilepsy do not get adequate seizure control with the current medications (Schmidt and Sillanpää, 2012). Thus, there is an urgent need for more effective (and better tolerated) treatments to control drug-resistant seizures, as well as for innovative therapies to prevent, stop or reverse the development of epilepsy in at-risk individuals (Galanopoulou et al., 2012).

In principle, understanding the molecular mechanisms underlying the neurobiological alterations occurring during epileptogenesis and finding ways to manipulate them should allow development of effective agents. Although the epileptogenic process remains incompletely understood, recent molecular studies began to elucidate the mechanisms that regulate some components of the circuitry reorganizations (including cell death, axonal and dendritic plasticity, neurogenesis and functional alterations in ion channel and synaptic properties) that occur during epileptogenesis and likely contribute to the development of hyperexcitability and spontaneous seizures (Pitkänen and Lukasiuk, 2011). Microarray-based gene expression studies indicate that products of genes regulated during epileptogenesis belong to a variety of functional classes, including signal transduction, transcription regulation, neurogenesis and immune response proteins (Pitkänen and Lukasiuk, 2009).

Which of these many molecular changes should be a target for intervention? Neurotrophic factors (NTFs) appear to be very strong candidates, because an extensive literature demonstrates their involvement in each of the above-mentioned cellular alterations associated with epileptogenesis (Simonato et al., 2006; Simonato and Zucchini, 2010): not only their trophic effects suggest an involvement in cell death, neurogenesis and axonal sprouting, but they also exert functional effects at the synaptic level, with distinct modulatory actions at excitatory and inhibitory synapses (Schinder and Poo, 2000). Furthermore, NTFs are greatly involved in brain development, and epileptogenesis is thought to recapitulate several aspects of developmental processes (Kim et al., 2010; Simonato and Zucchini, 2010; Ueda et al., 2011).

Identification of the specific roles played by NTF families or even single NTFs in the morpho-functional changes associated with epileptogenesis is very difficult. It seems likely that multiple NTFs are involved in the process at distinct phases and with distinct roles (Simonato et al., 2006). Nonetheless, a few specific molecules have gained particular interest and attention, like the brain-derived neurotrophic factor (BDNF) and members of the fibroblast growth factor (FGF) family, especially FGF2 (Simonato et al., 2006). In fact, the combined supplementation of BDNF and FGF2 in the epileptogenic area during the latency period between an epileptogenic insult and the first spontaneous seizure has been reported to produce a dramatic attenuation of the adaptive morpho-functional changes occurring in the tissue (namely cell loss, aberrant neurogenesis, sprouting of the mossy fibers—i.e., of hippocampal granule cell axons—and neuroinflammation), ultimately leading to reduced frequency and severity of spontaneous seizures, i.e., a disease-modifying and maybe truly anti-epileptogenic effect (Paradiso et al., 2009; Bovolenta et al., 2010; Paradiso et al., 2011; Simonato et al., 2013).

These very promising results prompt further investigation of the role played in epileptogenesis by neurotrophins like BDNF and FGFs like FGF2. Here, we will focus on the involvement of FGF family members in the morpho-functional alterations associated with epilepsy. We will first summarize the biological features of this class of NTF and then describe existing evidence supporting their role in epileptic disorders and specifically in epileptogenesis.

## The FGFs

The human FGFs contain 150–300 amino acids and have a conserved core of 120 amino acids with 30–60% identity. The family encompassed 18 members. FGF15 has not been identified in humans and FGF19 has not been identified in mice and rats, thus it has been hypothesized that they are the products of orthologous genes. Four previously listed members, now termed FGF homologous factors (FHF1-4), have been removed from the original list of 23 (Goldfarb et al., 2007) because they exert purely intracrine effects. FHF1, FHF2, FHF3 and FHF4, which in the old nomenclature correspond respectively to FGF12, FGF13, FGF11 and FGF14, are not secreted extracellularly and act intracellularly in an FGF receptor-independent manner. They interact with intracellular domains of voltage gated sodium channels and with a neuronal mitogen-activated protein kinase (MAPK) scaffold protein, islet-brain-2. FGF homologous factor (FHFs) are thought to be mainly active in postnatal life, and their only known role is in the regulation of excitability by association with sodium channels (Itoh, 2010).

The currently classified 18 members of the mammalian FGF family can be functionally sub-divided into canonical FGFs and hormone-like FGFs (hFGFs) based on their paracrine or endocrine actions (Itoh and Ornitz, 2011). Both sub-groups mediate biological responses in an FGF receptor-dependent manner, but hFGF can act over long distances like endocrine hormones. Most FGFs are secreted proteins with cleavable N-terminal secretion signal sequences. In this respect, FGF1 and FGF2 are atypical, because they do not have these N-terminal sequences, but they may nonetheless be released from damaged cells or via exocytotic mechanism(s) independent from the endoplasmic reticulum-Golgi pathway.

Almost all FGF effects are mediated by binding to cell surface tyrosine kinase receptors (FGFRs). Heparin/heparan sulfate acts as a cofactor for the binding of FGFs to FGFRs. FGF1, FGF2 and FGF3 can also directly translocate to the nucleus and act in an intracrine manner (Itoh and Ornitz, 2011). Four genes, *FGFR1-FGFR4*, have been identified in humans and mice that encode for high-affinity FGFRs (FGFR1 through FGFR4). These genes display substantial sequence homology and are similar in their general structure: all are tyrosine kinase receptors with one membrane-spanning domain and an extracellular ligand-binding domain with three immunoglobulin-like motifs (Ig I, II and III). The Ig-like domain III is an essential determinant of ligand binding. *FGFR1-FGFR3* encode two main versions of this domain (termed IIIb and IIIc) generating, by alternative splicing, a total of six FGFR proteins (FGFR 1b, 1c, 2b, 2c, 3b, 3c), whereas *FGFR4* generates a single protein (FGFR4). FGF binding to FGFRs induces dimerization, receptor trans-phosphorylation and activation of four key downstream signaling pathways: RAS-RAF-MAPK, PI3K-AKT, STAT and PLCγ (Beenken and Mohammadi, 2009; Turner and Grose, 2010).

Heparan sulfate interacts with heparan binding sites in the FGFR Ig II domain and in the FGF molecule, favoring protein-protein contacts at the dimer interface and thereby sustaining dimerization. Dimerization enables tyrosine trans-phosphorylation of the intracellular kinase domains and generates docking sites for the recruitment and phosphorylation of downstream signaling substrates, ultimately leading to alterations in expression of specific genes and to the biological effects. The acid box-containing linker between Ig domains I and II may serve as auto-inhibitory control on heparan sulfate-dependent receptor dimerization (Kalinina et al., 2012). The endocrine hFGFs (FGF15/19, FGF21 and FGF23) bind to FGFRs and heparin/heparan sulfate with very low affinity (Itoh, 2010). FGF23 activates FGFR1c, which forms a complex with α-Klotho, a single-pass transmembrane protein of 1,000 amino acids with a short cytoplasmic domain predominantly expressed in the kidney, parathyroid glands, and epithelial cells of choroid plexuses in the brain. Similarly, FGF15/19 can bind to FGFR4 and β-Klotho, a protein structurally and functionally similar to α-Klotho that is predominantly expressed in the liver, pancreas and adipose tissue.

## FGFs in epilepsy

**Figure 1:**
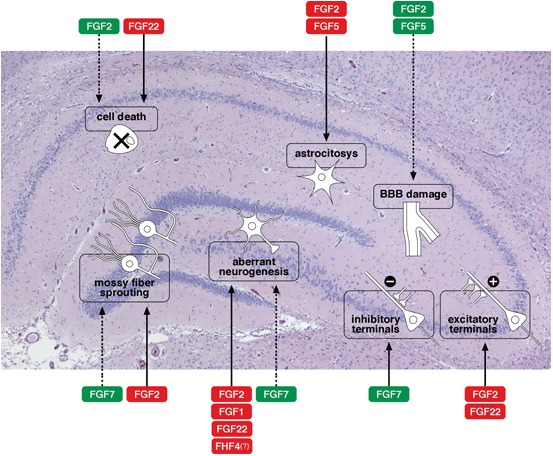
**Effects of FGFs on morpho-functional alterations associated with epileptogenesis in the hippocampus.** A schematic representation of some of the best-characterized effects of FGFs on the morpho-functional changes associated with epileptogenesis in the hippocampus: cell death, astrocytosis, blood-brain barrier (BBB) damage, alterations in synaptogenesis (density of excitatory or inhibitory terminals), axonal sprouting (like sprouting of the mossy fibers), aberrant neurogenesis with newborn neurons in the dentate gyrus hilus. Solid arrows indicate facilitation, dotted arrows inhibition of a specific event. The name of the FGF members is framed in red to indicate a putatively negative implication in epilepsy (favoring epileptogenesis), in green to indicate a putatively positive implication (contrasting epileptogenesis). Note that these are only tentative indications, because evidence regarding the FGFs is often fragmentary and because the patho-physiological consequences of each alteration are sometimes uncertain.

The production and release of many members of the FGF family have been reported to be altered (increased in most cases) in epilepsy: these include FGF1, −2 and −5 (Gómez-Pinilla et al., 1995; Riva et al., 1995; Cuevas and Giménez-Gallego, 1996; Simonato et al., 1998; Bregola et al., 2000), FGF7 and −22 (Terauchi et al., 2010; Lee et al., 2012) FGF8 and −17 (McCabe et al., 2011; Zanni et al., 2011), as well as FHF4 (Hu et al., 2011) and α-Klotho (Park et al., 2009). These observations prompted investigations on possible functional roles played by the FGFs in epileptic models and human syndromes. At present, data are still rather fragmentary and incomplete. Figure 1 provides a schematic representation of some of the best-characterized effects of FGFs on the morpho-functional changes associated with epileptogenesis in the hippocampus. The member on which available information is most robust and convincing is FGF2 (Zucchini et al., 2005, 2008), that will be therefore discussed separately and in greater detail.

Research on FGFs involvement in epilepsy has been conducted mainly in three types of models. First, acute seizure models in which convulsions are produced in normal animals. These include maximal electroshock (MES) and administration of convulsant agents. Second, kindling: a model in which the repeated administration to a discrete limbic brain area of an initially subconvulsive electrical stimulation induces seizures that progressively intensify in duration and severity, from focal to secondarily generalized. Kindling can be evoked by stimulating different areas, including the amygdala, hippocampus, piriform cortex. Third, chemically (pilocarpine or kainate) or electrically (self-sustained SE) evoked SE: these are models in which induction of an epileptogenic insult SE is followed by a latency period during which the animals are apparently well and then by spontaneous recurrent seizures (SRSs), i.e., epilepsy. This situation most closely mimics the one occurring in humans with acquired epilepsies.

### FGF1

FGF1 (also known as acidic FGF, aFGF) has been reported to be implicated in rodent models of epilepsy but, to date, it has not been studied in the human disease (Naegele, 2009). MES in rodents can produce epigenetic modifications of DNA leading to increased neurogenesis. This phenomenon appears to be FGF1-dependent and related to *Gadd45b*, a gene involved in DNA repair and DNA 5-methylcytosine excision (Ma et al., 2009): Gadd45b is responsible for demethylating regulatory promoter regions in the genes encoding for FGF1 and BDNF. However, the role in epilepsy of neurogenesis (and, based on these studies, of FGF1) is still uncertain. Many consider it as a maladaptive plastic change (Parent, 2007; Jung et al., 2009; Naegele, 2009; Hattiangady and Shetty, 2010), implicating a pro-epileptic role for FGF1. In contrast with this view, however, systemic administration of the recombinant human FGF1 has been reported to exert anticonvulsant effects in kainate-induced convulsions and mortality (Cuevas and Giménez-Gallego, 1996).

### FGF5

Increased expression of FGF5 has been found shortly after acute seizures, in association with an increased transcription of *FGF2* and *FGFR1* (Gómez-Pinilla et al., 1995). In contrast with FGF2, whose constitutive expression is mainly in astrocytes (see below), FGF5 is mostly found in neurons. Similar to FGF2 knock-out mice, however, decreased expression levels of the intermediate filament component glial fibrillary acidic protein (GFAP) has been reported in FGF5 deficient mice, even if the density of astrocytes remains unchanged (Reuss et al., 2003). FGF5 has been hypothesized to favor astrocyte proliferation, based on the observation that FGF5 and its high-affinity receptor FGFR1 IIIc are overexpressed in astrocytic brain tumors (Allerstorfer et al., 2008). Whether this effect also occurs in epileptogenesis and favors epilepsy-associated astrocytosis is still unknown. Astrocytosis contribute to induce many pathological events associated with epileptogenesis, including increased inflammation and neuronal hyperexcitability (Vezzani et al., 2011; Kim et al., 2012).

In functional contrast with these putative effects on astrocytosis, FGF5 may contribute to the stabilization of the blood—brain barrier (BBB). A reduction of GFAP protein levels has been observed in the perivascular astroglial endfeet of FGF5 (and FGF2) deficient mice (Reuss et al., 2003), resulting in increased BBB permeability, an event associated with epileptogenesis. Therefore, FGF5 appears to exert contrasting effects in epilepsy development: on one hand, it may protect the BBB; on the other hand, it may favor reactive astrocytosis.

### FGF7 and FGF22

FGF22 and FGF7 have been reported to promote the organization of excitatory and inhibitory presynaptic terminals, respectively, as target-derived presynaptic organizers. These factors are target-derived molecules that promote differentiation of neuritic segments into presynaptic nerve terminals. FGF22 and FGF7 are expressed in the CA3 pyramidal neurons of the rodent and human (at least FGF22) hippocampus (Umemori et al., 2004; Katoh and Katoh, 2005; Terauchi et al., 2010; Lee et al., 2012). The formation of excitatory or inhibitory synaptic contacts on dendrites of hippocampal CA3 pyramidal neurons is specifically impaired in mice lacking FGF22 or FGF7, respectively. Specifically, the clustering of vesicles containing the excitatory neurotransmitter glutamate is impaired in FGF22 deficient mice, whereas the clustering of inhibitory, GABA-containing vesicles is reduced in FGF7 deficient mice (Jones and Basson, 2010). These presynaptic defects are rescued by postsynaptic expression of the appropriate FGF, demonstrating that there is an absolute requirement of FGF7 during the formation of inhibitory GABAergic synapses and of FGF22 during excitatory glutamatergic synaptogenesis. The differential effects of FGF22 and FGF7 are likely due to distinct synaptic localizations and employment of different signaling pathways.

The implications for epilepsy are of course opposite. FGF22 knock-out mice (with reduced excitatory synapses) are resistant to epileptic seizures, whereas FGF7 knock-out mice (with reduced inhibitory synapses) are prone to seizures (Terauchi et al., 2010; Lee et al., 2012). In addition, increased neurogenesis and mossy fiber sprouting have been reported in FGF7 knock-out mice during post-SE epileptogenesis, both events that may also favor susceptibility to epilepsy development in these mice (Lee et al., 2012). Therefore, FGF7 activation may be capable of decreasing vulnerability to epilepsy by multiple mechanisms. In contrast, epileptogenesis-associated aberrant neurogenesis and cell death in the hippocampal dentate gyrus hilus are suppressed in FGF22 knock-out mice (Lee and Umemori, 2013), suggesting that inhibition of FGF22 may alleviate epileptogenesis.

### FGF8 and FGF17

FGF8 and FGF17 are implicated in two epileptogenic human neurological disorders. Recently, *FGF8* gene mutations have been identified in recessive holoprosencephaly and in septo-optic dysplasia (Moebius syndrome), and *FGF17* gene chromosomic deletion in Dandy–Walker malformation (McCabe et al., 2011; Zanni et al., 2011). In all these diseases, patients can experience spontaneous seizures.

### FHF4

The intracrine FHF4 (FGF14 according to the old nomenclature) also seems to be involved in the morpho-functional alterations associated with epilepsy. De-repression of *FGF14* gene expression is obtained in conditional neuron-restrictive silencer factor (NRSF) knock-out mice (Hu et al., 2011). In fact, the degree of up-regulation of FHF4 following kainate-induced SE is significantly increased in the cortex of NRSF knock-out mice compared with controls. In the kindling model, these mice exhibit dramatically accelerated seizure progression, prolonged after-discharge duration and increased mossy fiber sprouting compared with controls. Thus, FHF4 appears to favor epileptogenesis. The mechanism of this effect remains unknown, but it can be hypothesized that it depends on increased neurogenesis and/or synaptogenesis (Wang et al., 2002; Hu et al., 2011).

### α-Klotho

Pathological activation of α-Klotho may be implicated in phenylketonuria (PKU), an autosomal recessive disorder caused by a deficiency of phenylalanine hydroxylase, an enzyme that catalyzes the conversion of phenylalanine to tyrosine. The resultant hyper-phenylalaninemia causes mental retardation, seizures, and abnormalities in behavior and movement. The mechanism of this disease remains incompletely understood, but an implication of the FGF family has been proposed based on the observation of an α-Klotho-dependent increase in Na^+^/K^+^-ATPase activity (Park et al., 2009; Itoh, 2010).

## FGF2 and epilepsy

### Expression in the brain

FGF2 (basic FGF, bFGF) expression is developmentally regulated. It is highly expressed in neurons in the fetal brain and, later in development, in glia cells (Caday et al., 1990; Torelli et al., 1990); its levels increase progressively in early postnatal life, and then remain high in the adult and aged rat brain (Riva and Mocchetti, 1991).

### Biological activities

FGF2 is thought to play a critical role in cell-cell signaling between neurons, astrocytes and microglia during development (Gremo and Presta, 2000). In adults, FGF2 can regulate proliferation of neural stem cells and neuronal survival (Bikfalvi et al., 1997; Hefti, 1997). Furthermore, it enhances axonal branching (Waliche, 1988; Aoyagi et al., 1994; Abe et al., 2001; Szebenyi et al., 2001) and synaptogenesis (Li et al., 2002) in neurons.

FGF2 exerts neuroprotective effects against a wide variety of insults, reducing brain cellular damage and improving functional recovery in experimental models of stroke, epilepsy, traumatic brain and spinal cord injury (Liu and Holmes, 1997a; Teng et al., 1999; Li and Stephenson, 2002; Zucchini et al., 2008). This neuroprotective effect seems to depend on interference with a number of signaling pathways, including expression and gating of N-methyl-D-aspartate (NMDA) receptors, maintenance of Ca^2+^ homeostasis, regulation of reactive oxygen species (ROS) detoxifying enzymes and strengthening of anti-apoptotic pathways (Acharya et al., 2008).

*In vitro*, FGF2 increases survival, proliferation and differentiation of hippocampal neurons (Vicario-Abejon et al., 1995; Lowenstein and Arsenault, 1996). Interestingly, low FGF2 levels predominantly lead to the generation of neurons, whereas high levels generate glia and neurons (Vescovi et al., 1993; Qian et al., 1997). Similar effects are also observed *in vivo*. Neurogenesis is inhibited by injection of an anti-FGF2 antibody and increased by FGF2 injection in P1 rats (Tao et al., 1997; Cheng et al., 2002). The effects of exogenous FGF2 is also observed in the adult brain, in areas of constitutive neurogenesis, i.e., subgranular zone of the dentate gyrus and subventricolar zone (Wagner et al., 1999). FGF2 is the most potent known mitotic agent for adult neural stem and progenitor cells. Moreover, it regulates astroglial cell differentiation, functions, and transition to the “reactive” phenotype observed after lesions (Reuss et al., 2003). As in FGF5 deficient mice, a reduction of GFAP protein levels has been observed in the perivascular astroglial endfeet of FGF2 knock-out mice (Reuss et al., 2003), which results in increased BBB permeability.

FGF2 accelerates bifurcation and growth of axonal branches in cultured rat hippocampal neurons (Aoyagi et al., 1994). A greater number of axon branches is expected to produce a greater number of synaptic contacts and, indeed, local application of FGF2 has been found to increase the number of morphologically mature and functionally active excitatory synapses between hippocampal neurons (Li et al., 2002).

### FGF2 and epilepsy

Based on its biological properties, FGF2 seems particularly likely to be involved in epileptogenesis. Indeed, (1) seizures increase FGF2 mRNA and protein levels in specific brain areas and up-regulate the expression of FGFR1 receptors; (2) acute intra-hippocampal injection of FGF2 causes seizures, while chronic i.c.v. infusion of low dose FGF2 does not affect kainate seizures but promotes behavioral recovery and reduces hippocampal damage; (3) kainate seizure severity is not altered in FGF2 knock-out mice, but is increased in FGF2 over-expressing mice.

FGF2 gene expression is induced with similar patterns in different acute seizure models (Riva et al., 1992; Bugra et al., 1994; Follesa et al., 1994; Gall et al., 1994; Riva et al., 1994; Gómez-Pinilla et al., 1995; Kondratyev et al., 2002). This phenomenon is fast, marked and transient, peaking at 6–24 h in different hippocampal subfields and in the cortex. In the hippocampus of naive rats, FGF2 is expressed diffusely in astrocytes and in CA2 pyramidal neurons (Ernfors et al., 1990; Gomez-Pinilla et al., 1992; Woodward et al., 1992). Following acute seizures, increased *FGF2* mRNA levels in these cell populations, as well as new expression in CA1 pyramidal neurons and in dentate gyrus granule cells have been observed (Gall et al., 1994; Riva et al., 1994). In the kindling model, induction of *FGF2* mRNA expression is observed in a more pronounced manner after a single after-discharge, not accompanied by behavioral seizures, than after a fully kindled, generalized tonic-clonic seizure lasting more than a minute (Simonato et al., 1998; Bregola et al., 2000). In addition, FGF2 expression in limbic regions has been reported to be more pronounced after partial than after generalized electroshock seizures (Follesa et al., 1994). These observations suggest that the duration and intensity of seizures within a specific area does not necessarily correlate with the magnitude of *FGF2* mRNA level increase, and that FGF2 may be more directly implicated with epileptogenesis than with generalized seizure expression. Induction of mRNA for FGF2 is typically followed by an increase in FGF2 protein: FGF2-like immunoreactivity is detectable 6 h following seizures, peaks after about 24 h and may remain elevated up to 30 days, being mainly localized in the nuclei of astrocytes (Humpel et al., 1993; Gómez-Pinilla et al., 1995; Ballabriga et al., 1997; Gwinn et al., 2002). Increased levels are mainly observed for the high molecular weight isoforms of FGF2, which contain nuclear targeting sequences, and therefore may enter the nucleus and influence gene regulation, activating programs for cellular plasticity or proliferation (Gwinn et al., 2002). Therefore, the transient pattern of *FGF2* mRNA elevation may have prolonged translational effect influencing long-term plasticity changes. Finally, seizure-induced increases in the neuronal and astrocytic expression of a high-affinity FGF receptor (FGFR1) have been found in an epilepsy model (Bugra et al., 1994; Van Der Wal et al., 1994; Gómez-Pinilla et al., 1995). A strong FGFR3 staining has also been found in reactive microglia in several brain areas, including the hippocampus, 30 days after kainate injection (Ballabriga et al., 1997). These observations lead to the notion that epileptogenic seizures co-ordinately increase the expression of FGF2 and of its receptor(s). The hypothesis that these events may take part in the plastic changes associated with epilepsy has been pharmacologically and genetically investigated.

Injection of FGF2 into the dentate region of the ventral hippocampus causes an immediate excitatory effect culminating in EEG and behavioral seizures (Liu and Holmes, 1997b). In contrast, the chronic infusion of low FGF2 doses into the cerebral ventricles does not modify latency and duration of kainate seizures, but prevents seizure-induced hippocampal cell loss and improves long-term behavioral recovery (Liu et al., 1993; Liu and Holmes, 1997a). This neuroprotective action may depend on the induction of activin A (ActA), a cytokine belonging to the transforming growth factor-beta superfamily. When co-injected with kainate in the hippocampus, FGF2 prevents the loss of CA3 neurons in mice. In mice treated with kainate and FGF2, but not in those treated with kainate alone, ActA-immunoreactivity is high in pyramidal neurons (Tretter et al., 2000) and FGF2 fails to protect CA3 neurons against kainate-induced death in the presence of the ActA-neutralizing protein follistatin (Tretter et al., 2000).

Studies in FGF2 knock-out and transgenic mice have extended these pharmacological findings. The FGF2 knock-out mice (Ortega et al., 1998) are viable, fertile and without any obvious phenotypical difference from their wild-type littermates, but exhibit a significant reduction in the number of neurons in the neocortex and a delayed would healing. The susceptibility to seizures of FGF2 knock-out mice has been studied in the kainate model (Yoshimura et al., 2001). The severity of kainate seizures did not differ between knock-out and wild-type mice. However, while an increase in FGF2 protein levels and neuroproliferation was observed in the hippocampus of wild-type mice, very low levels of neurogenesis were observed in the knock-outs (Yoshimura et al., 2001). High FGF2 levels were restored in the mutant mice using a viral gene delivery system, leading to levels of neurogenesis comparable to those of wild-type littermates. These data suggest that seizure-induced FGF2 overexpression is necessary and sufficient to prime proliferation of neural progenitor cells in the adult hippocampus (Yoshimura et al., 2001).

In an attempt to further elucidate the effect of FGF2 in epilepsy, we studied transgenic mice (TgFGF2) expressing the human FGF2 (Coffin et al., 1995; Fulgham et al., 1999). By gross examination, these mice are affected by skeletal malformations, such as shortening and flattening of long bones and moderate macrocephaly (Coffin et al., 1995). In addition, without having any spontaneous vascular defect, they exhibit a predisposition to angiogenic reactions with subsequent amplified angiogenesis (Fulgham et al., 1999). TgFGF2 mice display increased FGF2 expression in hippocampal pyramidal neurons and dentate granule cells. Increased density of glutamatergic synaptic vesicles is observed in the hippocampus, and electrophysiological data confirm an increase in excitatory inputs to CA1, suggesting the presence of a latent hyperexcitability (Zucchini et al., 2008). Indeed, TgFGF2 mice display increased susceptibility to kainate-induced seizures compared with wild-type littermates, in that latency to generalized seizure onset is reduced, while behavioral seizure scores and lethality are increased. Wild-type and TgFGF2 mice with similar seizure scores were employed for examining seizure-induced cellular consequences. Neurogenesis and mossy fiber sprouting are not significantly different between the two groups. By contrast, cell damage is significantly lower in TgFGF2 mice, especially in the areas of FGF2 overexpression (CA1 and CA3), indicating reduction of seizure-induced necrosis and apoptosis. These data are in good agreement with the neuroprotective action of FGF2 in injury models, and it can be hypothesized (as above) that seizures prompt the over-production of FGF2 in astrocytes and neurons and, in turn, this newly produced FGF2 enhances the production of ActA in selected neurons. In the hippocampus, ActA may reach high levels in CA1 and CA3 neurons, protecting these cell types from injury (Mattson, 2000). Accordingly, in FGF2 transgenic mice we observed the preservation from degeneration of CA1 and CA3 neurons, but not of those in the hilus of the dentate gyrus. Finally, we explored possible long-term synaptic rearrangements, like the sprouting of mossy fibers. Under control condition, neither wild-type of TgFGF2 mice display sprouting of the mossy fibers. Thirty days after kainate treatment, sprouting is observed in similar grade both in transgenic and in control mice (Zucchini et al., 2008).

Altogether, these data suggest that FGF2 may be implicated in seizure susceptibility and in seizure-induced plasticity, exerting different, and apparently contrasting effects: favoring acute seizures but reducing seizure-induced cell death. Coherent with this idea, FGF2 has been suggested to be implicated in the preconditioning effect of brief, non-injurious seizures that can protect against cell death induced by otherwise harmful insults, like adrenalectomy or kainate-induced SE (Kelly and McIntyre, 1994; Masco et al., 1999; Kondratyev et al., 2001). These apparently contrasting effects could depend on activation of different receptor subtypes (Simonato et al., 2006), a hypothesis not yet explored.

In addition to rodent studies, the implication of FGF2 has been investigated in human epilepsy-associated malformations of cortical development by autoptic analysis and corticectomy specimens. The data support the notion that FGF2 favors epilepsy development by altering gliogenesis and maturation of cortical neurons from migrating neuroblasts (Ueda et al., 2011).

## Conclusions

The data described in this review clearly demonstrate that the FGFs are highly involved in the generation of the morpho-functional alterations in brain circuitries associated with epileptogenesis, that will eventually lead to unbalanced control of excitability and spontaneous seizures, i.e., epilepsy. As noted, information is still very incomplete, with many FGF members still unexplored and with very limited information on the FGF receptors. Moreover, these findings should be integrated with others on the involvement of other NTF families (Simonato et al., 2006).

Altogether, it seems likely that the morphology and function of adult brain circuits is regulated in a highly redundant manner. With reference to epilepsy, the emerging picture is impressively complex: multiple NTFs implicated at different levels and in different areas, producing synergic or antagonistic effects on biological parameters that, in turn, may facilitate or oppose the transformation of a normal tissue in epileptic. Therefore, even if, in principle, identifying key elements in these phenomena may lead to effective therapies, reaching this goal will require confronting this huge complexity.

Dissecting out this situation is not trivial at all. One first step could be to generate a sort of “neurotrophicome” listing all NTFs (and NTF receptors) that are active during epileptogenesis. This would require identification of the extent to which each NTF is active (concentrations at the site of action); how it is active (local representation of receptor subtypes, including low-affinity receptors); when in the natural history of disease this occurs; how the NTF at hand will possibly interact with other NTFs. Once a sufficiently realistic picture of this “neurotrophicome” will become available, it will still be needed to determine a strategy for intervention and to develop appropriate tools to manipulate (and possibly correct) the pathological situation.

All this is extraordinarily challenging but, at the same time, is extremely fascinating and holds the promise of outstanding rewards: a better understanding of the brain function, and maybe a cure for epilepsy and other neurological diseases.

## Conflict of interest statement

The authors declare that the research was conducted in the absence of any commercial or financial relationships that could be construed as a potential conflict of interest.
